# Long-Term Efficacy and Safety of Risankizumab for Moderate to Severe Psoriasis: A 2-Year Real-Life Retrospective Study

**DOI:** 10.3390/jcm12093233

**Published:** 2023-04-30

**Authors:** Matteo Megna, Angelo Ruggiero, Teresa Battista, Laura Marano, Sara Cacciapuoti, Luca Potestio

**Affiliations:** Section of Dermatology—Department of Clinical Medicine and Surgery, University of Naples Federico II, Via Pansini 5, 80131 Napoli, Italy

**Keywords:** psoriasis, risankizumab, anti-IL23, biologics

## Abstract

Risankizumab is a humanized IgG monoclonal antibody inhibitor of IL23 and has been recently approved by the EMA and the FDA for the treatment of moderate to severe plaque psoriasis in adults who are candidates for systemic therapy. Its efficacy and safety have been reported by clinical trials and real-life studies. However, even if long-term data from trials have already been reported (up to 172 weeks), data on long-term real-life experiences are still limited. The aim of our study was to investigate the long-term (2 years) efficacy and safety of risankizumab for psoriasis management in a real-life setting. A monocentric retrospective study was performed, enrolling 168 patients affected by moderate to severe psoriasis who were undergoing treatment with risankizumab. Psoriasis severity and safety outcomes were evaluated at each follow-up visit (week 16, week 28, week 52, week 88, week 104). A statistically significant reduction of psoriasis severity scores was reported from week 16 and was maintained up to week 104. Moreover, interesting results in terms of safety have been collected, without any serious adverse events registered. Our long-term real-life monocentric retrospective study confirmed the efficacy and safety of risankizumab up to 104 weeks of treatment. However, further studies are required to confirm our results and to increase available data to establish the best evidence-based biologic selection algorithm.

## 1. Introduction

Psoriasis is a chronic inflammatory cutaneous disease affecting up to 3% of the worldwide population [1,2]. Psoriasis severity, together with the numerous comorbidities that may be associated with this disorder, may strongly affect the quality of life of patients and caregivers, hence the need for early and efficient treatment [3,4,5]. However, the management of moderate to severe psoriasis may be challenging [6,7]. Recently, the introduction of biologic drugs positively revolutionized the therapeutic landscape [8,9]. In particular, the knowledge on the pathogenetic crucial role of the interleukin (IL)-23/IL17 axis led to the development of selective efficacious therapies [10]. Biologics targeting the p40 subunit common to both IL12 and IL23 have shown good results, but selectivity for IL23p19 offers higher efficacy and safety performance with respect to anti-p40 blockade [11].

Risankizumab is a humanized IgG monoclonal antibody inhibitor of IL23 recently approved by the EMA and the FDA for the treatment of moderate to severe plaque psoriasis in adults who are candidates for systemic therapy; and alone or in combination with methotrexate for the management of active psoriatic arthritis (PsA) in adults who have had an inadequate response or who have been intolerant to one or more disease-modifying antirheumatic drugs [12].

The use of risankizumab for psoriasis management was first reported by several phase III studies (UltIMMa-1 and UltIMMa-2, IMMhance, IMMerge, and IMMvent) which revealed good results in terms of effectiveness and safety profile. Notably, risankizumab was shown to be statistically significantly superior to placebo (IMMhance), ustekinumab and placebo (UltIMMa-1 and UltIMMa-2), secukinumab (IMMerge), and adalimumab (IMMvent) [13,14,15,16].

Recent data from real-world experience confirmed trial results, indicating risankizumab as a valuable tool in multifailure patients as well [17,18,19,20,21]. However, even if long-term data from trials have already been reported (up to 172 weeks) [17,18,19,20,21], data on long-term real-life experiences are still limited. The aim of our study was to investigate the long-term (2 years) efficacy and safety of risankizumab for psoriasis management in a real-life setting.

## 2. Materials and Methods

A monocentric retrospective study was performed enrolling patients affected by moderate to severe psoriasis undergoing treatment with risankizumab and attending the Psoriasis Care Centre of Dermatology at the University Federico II of Naples from September 2020 to March 2023.

The aim of the study was to evaluate long-term risankizumab efficacy and safety in a real-life setting. Inclusion criteria were: presence of moderate to severe plaque psoriasis assessed by a dermatologist for at least 6 months and risankizumab treatment for at least 16 weeks. Exclusion criteria were: <18-year-old patients, concomitant systemic treatment for psoriasis, and erythrodermic palmoplantar or generalized pustular psoriasis. At baseline, the following data were collected: demographic (age, sex) and clinical features (psoriasis duration, duration of PsA (if present), psoriasis severity through Body Surface Area (BSA) and Psoriasis Area Severity Index (PASI), comorbidities, previous and current psoriasis treatment). Psoriasis severity (PASI and BSA) and treatment response (PASI90 and PASI100) were evaluated at each follow-up visit (week 16, week 28, week 52, week 88, week 104). Similarly, safety was assessed by registering adverse events (AEs) and routine blood tests (blood count with formula, transaminases, creatinine, azotemia, glycaemia, erythrocyte sedimentation rate, serum protein electrophoresis, C reactive protein, total cholesterol and triglycerides) at the same timepoints. Risankizumab was scheduled at labelled dosage for psoriasis (150 mg as a subcutaneous injection at week 0, week 4, and every 12 weeks thereafter). The present study was conducted in accordance with the Declaration of Helsinki, and all patients gave their written informed consent before starting the study.

### Statistical Analysis

Descriptive statistics were used to analyze clinical and demographic data, presenting continuous variables as mean ± standard deviation and using number and proportion of patients for categorical ones. Statistical analysis using GraphPad Prism 4.0 (GraphPad Software Inc., La Jolla, CA, USA) was used to evaluate the statistical significance of clinical response, using the chi-squared test and Student’s *t*-test to assess the statistical significance of the differences in values obtained at the different timepoints of treatment for quantitative and qualitative characteristics of the populations. *p* values < 0.05 were considered to be statistically significant.

## 3. Results

A total of 193 patients undergoing treatment with risankizumab and attending our department were screened. Among these, 171 (88.6%) subjects fulfilled the inclusion and exclusion criteria. However, data were available only for 168 (87.0%) patients (94 males—56.0%; mean age 53.4 ± 8.3 years; range 21–73 years; mean psoriasis duration 15.1 ± 6.7 years) who were enrolled.

Patients’ demographics and clinical features at baseline have been reported in Table 1.

The most frequent comorbidity reported in our cohort was hypertension (82 cases—48.8%). Globally, 41.7% (n = 70) of patients were affected by PsA, and hypertension was the most frequent comorbidity assessed in 48.8% (n = 82) of patients, followed by dyslipidemia in 44.0% (n = 74) and diabetes in 10.7% (n = 18) subjects, respectively (Table 1). Moreover, 70 (41.7%) patients were affected by PsA.

All of the patients had received at least one or more conventional systemic treatment, with methotrexate as the most common (122 patients—72.6%), followed by cyclosporine (86—51.2%) and acitretin (36—21.4%) (Table 1). Furthermore, 135 (80.4%) patients had previously not responded to at least one biologic drug (anti-tumor necrosis factor (TNF)α: 130 (77.4%); anti-IL12/23: 56 (33.3%); anti-IL17: 82 (48.8%); anti-IL23: 6 (3.6%)) (Table 1). Notably, 24 (14.3%) subjects were bio-naïve, having not received any biologic before starting risankizumab. Globally, week 16 was reached by all of the patients (168—100.0%), whereas 152 (90.5%), 118 (70.2%), 98 (58.3%), and 76 (45.2%) patients completed week 28, 52, 88, and 104 follow-up visits, respectively.

Baseline clinical assessment showed a mean PASI of 15.3 ± 5.9 and a mean BSA of 24.3 ± 12.1.

**Figure 1:**
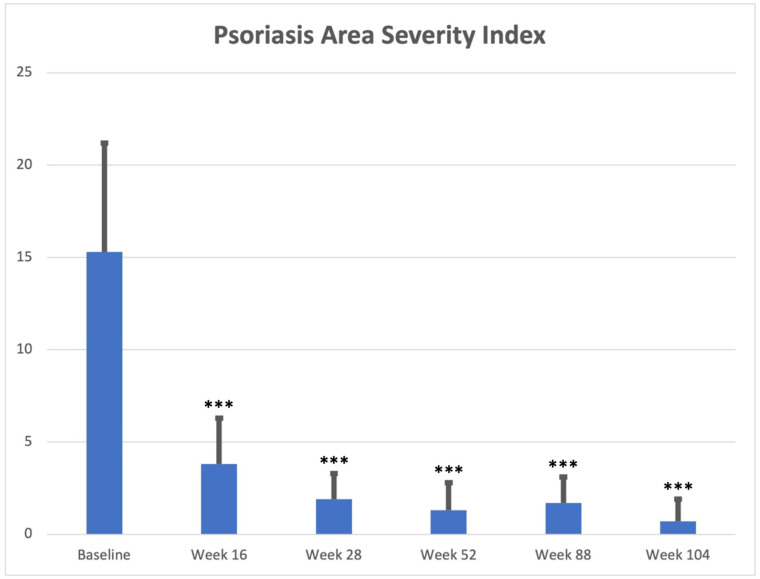
Mean PASI assessment (mean value and standard deviation) at baseline, week 16, week 28, week 52, week 88, and week 104. ***: *p* < 0.0001.

**Figure 2:**
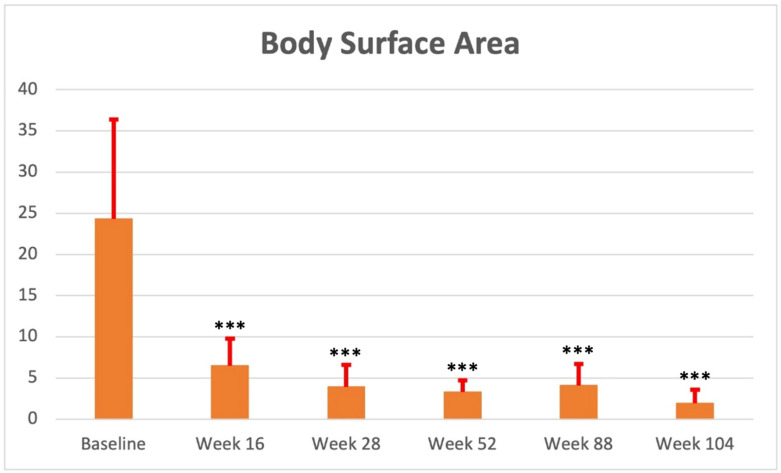
Mean BSA assessment (mean value and standard deviation) at baseline, week 16, week 28, week 52, week 88, and week 104. ***: *p* < 0.0001.

**Figure 3:**
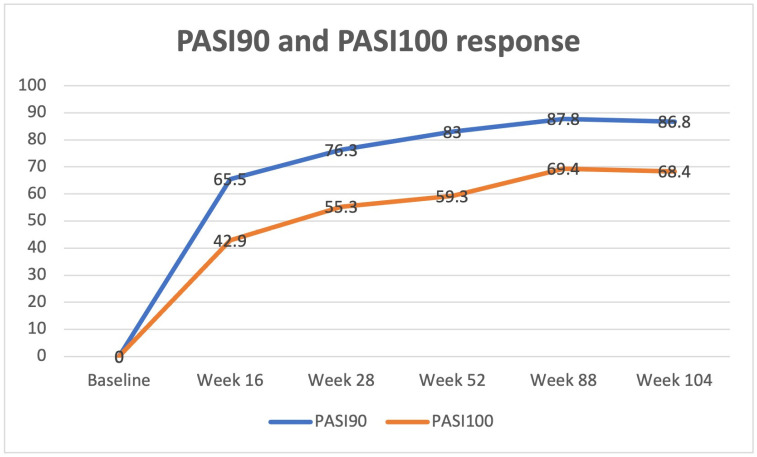
PASI90 and PASI100 response at week 16, week 28, week 52, week 88, and week 104.

A statistically significant improvement in both PASI and BSA was reported from week 16 (PASI: 3.8 ± 2.5; BSA: 6.5 ± 3.3 (*p* < 0.0001 for both)), with PASI90 and PASI 100 reached by 110 (65.5%) and 72 (42.9%) subjects, respectively. Therapeutic response was confirmed at each subsequent follow-up visit (e.g., week 52: PASI: 1.3 ± 1.5; BSA: 3.3 ± 1.4 (*p* < 0.0001 for both)) up to week 104 (PASI: 0.7 ± 1.2; BSA: 1.9 ± 1.7 (*p* < 0.0001 for both)). Clinical improvement was mostly maintained over time, with 98 (83.0%) and 70 (59.3%) subjects achieving PASI90 and PASI 100 at weeks 52 and 66 (86.8%) and 52 (68.4%) patients reaching the same scores at week 104, respectively. Psoriasis severity at baseline and each follow-up visit (with PASI90 and PASI100 response data) has been reported in Table 2 and Figure 1, Figure 2 and Figure 3.

Notably, sub-analysis regarding different factors (sex, comorbidities, body mass index, presence of PsA, bio-naïve patients vs. bio-experienced patients) that may positively or negatively affect therapeutic response did not show any statistically significant difference except in males, who were more correlated to a better therapeutic outcome in terms of PASI90 and PASI100 at week 16 compared with females (PASI90: 79.8% vs. 47.3%; PASI100: 54.3% vs. 28.4% (*p* < 0.05 for both)). Moreover, despite patients affected by PsA having a similar therapeutic response from week 52 compared with patients without PsA, these subjects showed a slightly lower clinical response (in terms of PASI90 and PASI100) at week 16 and week 28. Notably, these differences were not statistically significant.

Risankizumab discontinuation for inefficacy was reported in 15 (8.9%) subjects over the entire study period. However, no predictive factors for treatment withdrawal were assessed. Finally, (2.4%) patients were lost to follow-up (1 patient at week 28, 2 patients at week 52, 1 patient at week 88).

Regarding safety, no cases of serious AEs, injection site reaction, malignancy, or major cardiovascular events were registered in our study. However, mild AEs were reported in 48 (28.6%) subjects, with pharyngitis as the most common (22 patients—11.9%), followed by headache (13—7.7%) and flu-like illness (12—7.1%), none of whom required treatment interruption (Table 2). Furthermore, two cases of new-onset PsA were reported and successfully treated by adding methotrexate to risankizumab therapy.

Routine blood tests showed mild alterations in 28 (16.7%) subjects, as follows. Twelver patients (7.1%) showed mild transient hyperglycemia (range 127–147 mg/dL, n.v. 60–100 mg/dL); 7 patients (4.2%) showed hypertriglyceridemia (range 181–211 mg/dL, n.v. 45–175 mg/dL); and 9 patients (5.4%) showed an elevation of liver enzymes (5 patients: GPT—range 50–84 U/L, n.v. 0–46 U/L and GOT—range 78–120, n.v. 0–39 U/L; 4 patients: γ-GT—range 42–88 U/L, n.v. 11–40 U/L). None of these patients required treatment interruption.

Finally, 13 (7.7%) subjects precautionarily deferred risankizumab treatment (week range: 1–7) due to COVID-19 infection or for “at-risk” contact with a subject positive to SarsCov-2 infection.

## 4. Discussion

New advances in knowledge on the pathogenesis of psoriatic disease, in particular the crucial role of IL17/23 axis [10,11], have led to the development of new therapeutic tools with selective action that have made it possible to achieve significative results in terms of efficacy, with an optimal safety profile [22,23,24], even during the SARS-CoV-2 pandemic period [25,26]. To date, 12 biologic drugs have been approved for moderate to severe psoriasis [27,28], making real-life studies necessary to enable clinicians to choose a tailored approach for patients [29]. Among these, risankizumab, an innovative biologic drug selectively targeting IL23, showed highly significant results in terms of efficacy and safety in clinical trials [13,14,15,16]. Moreover, long-term data have been reported by a phase III, open-label extension study (LIMMitless) investigating the long-term effectiveness and safety of risankizumab in adult patients affected by moderate to severe plaque psoriasis. Globally, a total of 897 patients who completed precedent phase II/III trials (UltIMMa-1 and UltIMMa-2, IMMhance, SustaIMM, and NCT03255382) were enrolled. After 172 weeks of continuous treatment, PASI90 and PASI100 responses were reached by 85.5% and 54.4% of patients, respectively; moreover, rates of AEs remained stable and were comparable with those identified in the base studies [30]. Similarly, a long-term post hoc analysis of UltIMMa-1 and UltIMMa-2 confirmed that among 465 patients receiving risankizumab, more than 90% reached PASI ≤ 3 by week 172 and more than 80% achieved DLQI 0/1 [31].

However, despite real-life data confirming these results [17,18,19,20,21], they had a limited follow-up period; moreover, data on long-term real-life experiences are scarce, except for a 96-week multicenter experience [32]. In this scenario, we performed a long-term (2 years) study to investigate the effectiveness and safety of risankizumab for psoriasis management.

Our experience included 168 patients undergoing treatment with risankizumab for at least 16 weeks up to 104 weeks (n = 76—45.2%). In our cohort, both PASI and BSA significantly improved from the first follow-up visit at week 16 (PASI: 3.8 ± 2.5; BSA: 6.5 ± 3.3 (*p* < 0.0001 for both)), compared with baseline (PASI: 15.3 ± 5.9; BSA: 24.3 ± 12.1). Clinical response was maintained throughout the study (week 104: PASI: 0.7 ± 1.2; BSA: 1.9 ± 1.7 (*p* < 0.0001 for both)). Moreover, an interesting profile in terms of safety was assessed, with no cases of serious AEs and only 48 (28.6%) patients reporting mild AEs, none of whom required treatment interruption. 

Our results are in line with those of the longer real-life experience study by Adamczyk et al., who described the results of a multicenter study enrolling 185 patients evaluated after 4, 16, 28, 40, 52, and 96 weeks of treatment with risankizumab. Data at week 96 were available only for 22 patients, with 18 (81.8%) and 15 (68.2%) of these achieving PASI90 and PASI100 response, respectively [32]. As opposed to the authors, we did not find a significant negative correlation between PASI decrease and the presence of PsA, which seemed to be correlated to a slower therapeutic response up to week 52, without statistical significance [32]. Moreover, in our cohort, patient age and duration of psoriasis did not affect therapeutic outcome [32].

The remaining longer real-world studies are limited to 1 year of treatment. Ruiz-Villaverde et al. reported a PASI90 and PASI100 response at week 52 in 92.5% and 78.5% of patients in their cohort of 42 subjects [33]. Moreover, the authors noted that women had better efficacy results, suggesting female sex as a predictor of good response. Even if PASI90 and PASI100 responses are better than ours at week 52 (83.0% and 59.3%, respectively), we did not report female sex as a possible predictive factor of good response [33]. On the contrary, male sex seemed to be a predictive factor for a better clinical response, in our experience.

However, Hansel et al. also suggested female sex as a predictive factor of treatment response in their 52-week real-world experience, which enrolled 55 patients. Of these, 47 (85.5%) and 33 (60.0%) achieved PASI90 and PASI100 response by the end of the follow-up period [34].

Interestingly, Megna et al. reported the results of a 52-week real-life study enrolling 39 patients who had failed an anti-IL17 and who were undergoing treatment with risankizumab [35]. Globally, PASI90 and PASI100 were reached by 33 (84.6%) and 25 (64.1%) patients at week 52 [35]. In line with the authors, we found that switching from IL17 does not seem to affect risankizumab effectiveness [35]. Similarly, Caldarola et al. reported a PASI90 and PASI100 response in 95.24% (n = 60) and 90.48% (n = 57) subjects at week 52 in their cohort of 63 patients, suggesting that previous biological therapies did not influence the effectiveness of risankizumab [36]. Notably, the authors did not collect discontinuations related to AEs [36].

Total treatment discontinuation rates reported in real-life studies are very variable, ranging from 0% [33,37] up to 14.7% [36], with a mean of 5.8% [32,33,34,35,36,37,38,39].

Finally, the effectiveness and safety of risankizumab after 52 weeks of treatment have been also reported by Gargiulo et al. (131 patients: PASI90: 103, 78.6%; PASI100: 80, 61.1%; no severe AEs; no discontinuation for AEs) [37], Gkalpakiotis et al. (34 patients: PASI90: 28, 82.4%; PASI100: 23, 67.6%; no new safety issues; two permanent discontinuation for AEs—colorectal cancer and morbus Morbihan) [38], and Mastorino et al. (11 patients: PASI90: 82.%; PASI100: 73.%; no new safety issues; one treatment discontinuation for peri-malleolar edema) [39].

To sum up, risankizumab is an innovative drug among the armamentarium of available biologics for psoriasis management. Mid-term real-life studies confirmed its efficacy and safety, which were first revealed by clinical trials. However, long-term real-world data are still scarce. To the best of our knowledge, ours is one of the longest real-life experience studies investigating the effectiveness and safety of risankizumab in psoriasis management, surpassing the 96-week follow-up in the study of Adamczyk et al.

Our results suggest that clinical response during risankizumab treatment is maintained over time. However, further studies are needed to confirm our data.

## 5. Limitations of the Study

The retrospective design of the study and the limited number of enrolled patients who reached study end point may be the main limitations, reducing the generalizability of our results. The fact that 13 (7.7%) subjects temporarily suspended risankizumab scheduled administration due to COVID-19 infection or “at-risk” contact with a subject affected by COVID-19 may represent another limitation which could have influenced data. Notably, none of these patients reported a worsening of psoriasis during treatment suspension.

## 6. Conclusions

Our long-term real-life monocentric retrospective study confirmed the efficacy and safety of risankizumab up to 104 weeks of treatment. However, further studies are required to confirm our results and to increase available data to establish the best evidence-based biologic selection algorithm.

## Figures and Tables

**Table 1:** Patients’ demographics and clinical features at baseline.

**Patients, *n***	**168**
**Sex, M/F; *n (%)***	94/74 (56.0/44.0)
**Mean age *(years)***	53.4 ± 8.3
**Mean duration of psoriasis *(years)***	15.1 ± 6.7
**Psoriatic Arthritis, *n (%)***	70 (41.7%)
**Comorbidities, *n (%)***	
Hypertension	82 (48.8)
Diabetes	18 (10.7)
Cardiopathy	12 (7.1)
Dyslipidemia	74 (44.0)
Depression	10 (6.0)
Prostatic hyperplasia	4 (2.4)
Latent TB infection	6 (3.6)
Other	18 (10.7)
**Previous systemic conventional treatments, *n (%)***	
Cyclosporine	86 (51.2)
Methotrexate	122 (72.6)
Acitretin	36 (21.4)
Nb-UVB Phototherapy	32 (19.0)
**Previous biologic treatments, *n (%)***	
**Anti-TNFα**	130 (77.4)
Adalimumab	58 (34.5)
Etanercept	38 (22.6)
Infliximab	6 (3.6)
Certolizumab	20 (11.9)
Golimumab	8 (4.8)
**Anti-IL12/23**	56 (33.3)
Ustekinumab	56 (33.3)
**Anti-IL17**	82 (48.8)
Secukinumab	36 (21.4)
Ixekizumab	40 (23.8)
Brodalumab	6 (3.6)
**Anti-IL-23**	6 (3.6)
Guselkumab	4 (2.4)
Tildrakizumab	2 (1.2)
**Bio-naïve patients**	33 (19.6)

**Table 2:** Adverse events, discontinuation rate, and number of patients at each timepoint (week 16, week 28, week 52, week 88, week 104).

**Baseline: patients, *n* (%)**	**168 (100)**
**Week 16: patients, *n* (%)**	**168 (100.0)**
**Weeks 28: patients, *n* (%)**	**152 (90.5)**
**Week 52: patients, *n* (%)**	**118 (70.2)**
**Week 88: patients, *n* (%)**	**98 (58.3)**
**Week 104: patients, *n* (%)**	**76 (45.2)**
**Discontinuation rate for inefficacy, *n* (%)**	**15 (8.9)**
**Adverse events, *n (%)***	**48 (28.6)**
Pharyngitis	20 (11.9)
Flu-like illness	13 (7.7)
Headache	12 (7.1)
Diarrhea	8 (4.8)
Others	5 (3.0)

## Data Availability

The authors confirm that the data supporting the findings of this study are available within the article.

## References

[1] Raharja A., Mahil S.K., Barker J.N. (2021). Psoriasis: A brief overview. Clin. Med..

[2] Armstrong A.W., Read C. (2020). Pathophysiology, Clinical Presentation, and Treatment of Psoriasis: A Review. JAMA.

[3] Ruggiero A., Fabbrocini G., Cacciapuoti S., Cinelli E., Gallo L., Megna M. (2021). Ocular Manifestations in Psoriasis Screening (OcMaPS) Questionnaire: A Useful Tool to Reveal Misdiagnosed Ocular Involvement in Psoriasis. J. Clin. Med..

[4] Megna M., Ocampo-Garza S.S., Potestio L., Fontanella G., Gallo L., Cacciapuoti S., Ruggiero A., Fabbrocini G. (2021). New-Onset Psoriatic Arthritis under Biologics in Psoriasis Patients: An Increasing Challenge?. Biomedicines.

[5] Korman N.J. (2020). Management of psoriasis as a systemic disease: What is the evidence?. Br. J. Dermatol..

[6] Megna M., Potestio L., Fabbrocini G., Camela E. (2022). Treating psoriasis in the elderly: Biologics and small molecules. Expert Opin. Biol. Ther..

[7] Tokuyama M., Mabuchi T. (2020). New Treatment Addressing the Pathogenesis of Psoriasis. Int. J. Mol. Sci..

[8] Megna M., Camela E., Battista T., Genco L., Martora F., Noto M., Picone V., Ruggiero A., Monfrecola G., Fabbrocini G. (2023). Efficacy and safety of biologics and small molecules for psoriasis in pediatric and geriatric populations. Part I: Focus on pediatric patients. Expert Opin. Drug Saf..

[9] Megna M., Camela E., Battista T., Genco L., Martora F., Noto M., Picone V., Ruggiero A., Monfrecola G., Fabbrocini G. (2023). Efficacy and safety of biologics and small molecules for psoriasis in pediatric and geriatric populations. Part II: Focus on elderly patients. Expert Opin. Drug Saf..

[10] Ghoreschi K., Balato A., Enerbäck C., Sabat R. (2021). Therapeutics targeting the IL-23 and IL-17 pathway in psoriasis. Lancet.

[11] Puig L. (2017). The role of IL 23 in the treatment of psoriasis. Expert Rev. Clin. Immunol..

[12] Gu C., Yang J. (2019). Risankizumab for the treatment of psoriasis. Expert Rev. Clin. Pharmacol..

[13] Blair H.A. (2020). Risankizumab: A Review in Moderate to Severe Plaque Psoriasis. Drugs.

[14] Warren R., Blauvelt A., Poulin Y., Beeck S., Kelly M., Wu T., Geng Z., Paul C. (2020). Efficacy and safety of risankizumab vs. secukinumab in patients with moderate-to-severe plaque psoriasis (IMMerge): Results from a phase III, randomized, open-label, efficacy–assessor-blinded clinical trial. Br. J. Dermatol..

[15] Reich K., Gooderham M., Thaçi D., Crowley J.J., Ryan C., Krueger J.G., Tsai T.-F., Flack M., Gu Y., A Williams D. (2019). Risankizumab compared with adalimumab in patients with moderate-to-severe plaque psoriasis (IMMvent): A randomised, double-blind, active-comparator-controlled phase 3 trial. Lancet.

[16] Gordon K.B., Strober B., Lebwohl M., Augustin M., Blauvelt A., Poulin Y., A Papp K., Sofen H., Puig L., Foley P. (2018). Efficacy and safety of risankizumab in moderate-to-severe plaque psoriasis (UltIMMa-1 and UltIMMa-2): Results from two double-blind, randomised, placebo-controlled and ustekinumab-controlled phase 3 trials. Lancet.

[17] Megna M., Fabbrocini G., Ruggiero A., Cinelli E. (2020). Efficacy and safety of risankizumab in psoriasis patients who failed anti-IL-17, anti-12/23 and/or anti IL-23: Preliminary data of a real-life 16-week retrospective study. Dermatol. Ther..

[18] Megna M., Tommasino N., Potestio L., Battista T., Ruggiero A., Noto M., Fabbrocini G., Genco L. (2022). Real-world practice indirect comparison between guselkumab, risankizumab, and tildrakizumab: Results from an Italian 28-week retrospective study. J. Dermatol. Treat..

[19] Megna M., Cinelli E., Gallo L., Camela E., Ruggiero A., Fabbrocini G. (2022). Risankizumab in real life: Preliminary results of efficacy and safety in psoriasis during a 16-week period. Arch. Dermatol. Res..

[20] Sotiriou E., Bakirtzi K., Papadimitriou I., Tsentemeidou A., Eftychidou P., Eleftheriadis V., Lallas A., Ioannides D., Vakirlis E. (2021). A head-to-head comparison of risankizumab and ixekizumab for genital psoriasis: A real-life, 24-week, prospective study. J. Eur. Acad. Dermatol. Venereol..

[21] Borroni R.G., Malagoli P., Gargiulo L., Valenti M., Pavia G., Facheris P., Morenghi E., Di Corteranzo I.G., Narcisi A., Ortoncelli M. (2021). Real-life Effectiveness and Safety of Risankizumab in Moderate-to-severe Plaque Psoriasis: A 40-week Multicentric Retrospective Study. Acta Derm.-Venereol..

[22] Megna M., Potestio L., Camela E., Fabbrocini G., Ruggiero A. (2022). Ixekizumab and brodalumab indirect comparison in the treatment of moderate to severe psoriasis: Results from an Italian single-center retrospective study in a real-life setting. Dermatol. Ther..

[23] Ruggiero A., Potestio L., Cacciapuoti S., Gallo L., Battista T., Camela E., Fabbrocini G., Megna M. (2022). Tildrakizumab for the treatment of moderate to severe psoriasis: Results from a single center preliminary real-life study. Dermatol. Ther..

[24] Ruggiero A., Picone V., Martora F., Fabbrocini G., Megna M. (2022). Guselkumab, Risankizumab, and Tildrakizumab in the Management of Psoriasis: A Review of the Real-World Evidence. Clin. Cosmet. Investig. Dermatol..

[25] Marasca C., Annunziata M.C., Camela E., Di Guida A., Fornaro L., Megna M., Napolitano M., Patruno C., Potestio L., Fabbrocini G. (2022). Teledermatology and Inflammatory Skin Conditions during COVID-19 Era: New Perspectives and Applications. J. Clin. Med..

[26] Ruggiero A., Martora F., Picone V., Potestio L., Camela E., Battista T., Fabbrocini G., Megna M. (2022). The impact of COVID-19 infection on patients with psoriasis treated with biologics: An Italian experience. Clin. Exp. Dermatol..

[27] Dave R., Alkeswani A. (2021). An Overview of Biologics for Psoriasis. J. Drugs Dermatol..

[28] Kamata M., Tada Y. (2020). Efficacy and Safety of Biologics for Psoriasis and Psoriatic Arthritis and Their Impact on Comorbidities: A Literature Review. Int. J. Mol. Sci..

[29] Camela E., Potestio L., Fabbrocini G., Ruggiero A., Megna M. (2022). New frontiers in personalized medicine in psoriasis. Expert Opin. Biol. Ther..

[30] Papp K., Lebwohl M., Puig L., Ohtsuki M., Beissert S., Zeng J., Rubant S., Sinvhal R., Zhao Y., Soliman A. (2021). Long-term efficacy and safety of risankizumab for the treatment of moderate-to-severe plaque psoriasis: Interim analysis of the LIMMitless open-label extension trial beyond 3 years of follow-up. Br. J. Dermatol..

[31] Gooderham M., Pinter A., Ferris L., Warren R., Zhan T., Zeng J., Soliman A., Kaufmann C., Kaplan B., Photowala H. (2022). Long-term, durable, absolute Psoriasis Area and Severity Index and health-related quality of life improvements with risankizumab treatment: A post hoc integrated analysis of patients with moderate-to-severe plaque psoriasis. J. Eur. Acad. Dermatol. Venereol..

[32] Adamczyk M., Bartosińska J., Raczkiewicz D., Adamska K., Adamski Z., Czubek M., Kręcisz B., Kłujszo E., Lesiak A., Narbutt J. (2023). Risankizumab Therapy for Moderate-to-Severe Psoriasis—A Multi-Center, Long-Term, Real-Life Study from Poland. J. Clin. Med..

[33] Ruiz-Villaverde R., Rodriguez-Fernandez-Freire L., Pérez-Gil A., Font-Ugalde P., Galán-Gutiérrez M. (2022). Risankizumab: Efficacy, Safety, and Survival in the Mid-Term (52 Weeks) in Real Clinical Practice in Andalusia, Spain, According to the Therapeutic Goals of the Spanish Psoriatic Guidelines. Life.

[34] Hansel K., Zangrilli A., Bianchi L., Peris K., Chiricozzi A., Offidani A., Diotallevi F., Fargnoli M., Esposito M., Amerio P. (2021). A 52-week update of a multicentre real-life experience on effectiveness and safety of risankizumab in psoriasis. J. Eur. Acad. Dermatol. Venereol..

[35] Megna M., Potestio L., Ruggiero A., Camela E., Fabbrocini G. (2022). Risankizumab treatment in psoriasis patients who failed anti-IL17: A 52-week real-life study. Dermatol. Ther..

[36] Caldarola G., Zangrilli A., Bernardini N., Bavetta M., De Simone C., Graceffa D., Bonifati C., Faleri S., Giordano D., Mariani M. (2022). Risankizumab for the treatment of moderate-to-severe psoriasis: A multicenter, retrospective, 1 year real-life study. Dermatol. Ther..

[37] Gargiulo L., Ibba L., Pavia G., Vignoli C.A., Piscazzi F., Valenti M., Sanna F., Perugini C., Avagliano J., Costanzo A. (2022). Real-Life Effectiveness and Safety of Risankizumab in 131 Patients Affected by Moderate-to-Severe Plaque Psoriasis: A 52-Week Retrospective Study. Dermatol. Ther..

[38] Gkalpakiotis S., Cetkovska P., Arenberger P., Dolezal T., Arenbergerova M., Velackova B., Fialova J., Kojanova M. (2021). BIOREP study group Risankizumab for the Treatment of Moderate-to-Severe Psoriasis: Real-Life Multicenter Experience from the Czech Republic. Dermatol. Ther..

[39] Mastorino L., Susca S., Megna M., Siliquini N., Quaglino P., Ortoncelli M., Avallone G., Rubatto M., Fabbrocini G., Dapavo P. (2022). Risankizumab shows high efficacy and maintenance in improvement of response until week 52. Dermatol. Ther..

